# Association between Coffee Consumption/Physical Exercise and Gastric, Hepatic, Colon, Breast, Uterine Cervix, Lung, Thyroid, Prostate, and Bladder Cancer

**DOI:** 10.3390/nu13113927

**Published:** 2021-11-02

**Authors:** So Young Kim, Dae Myoung Yoo, Chanyang Min, Hyo Geun Choi

**Affiliations:** 1 Department of Otorhinolaryngology-Head & Neck Surgery, CHA Bundang Medical Center, CHA University, Seongnam 13496, Korea; sossi81@hanmail.net; 2 Hallym Data Science Laboratory, Hallym University College of Medicine, Anyang 14068, Korea; ydm1285@naver.com (D.M.Y.); joicemin@naver.com (C.M.); 3 Graduate School of Public Health, Seoul National University, Seoul 08826, Korea; 4 Department of Otorhinolaryngology-Head & Neck Surgery, Hallym University College of Medicine, Anyang 14068, Korea

**Keywords:** coffee, cancer, cohort studies, risk factors

## Abstract

Although the effects of coffee consumption and physical exercise on the risk of cancer have been suggested, their interactions have not been investigated. The present cross-sectional study aimed to investigate the correlation of coffee consumption and physical exercise with cancer. Participants ≥40 years old in the Korean Genome and Epidemiology Study 2004–2016 were included (*n* = 162,220). Histories of gastric cancer, hepatic cancer, colon cancer, breast cancer, uterine cervix cancer, lung cancer, thyroid cancer, prostate cancer, and bladder cancer were analyzed according to the coffee consumption groups using logistic regression models. The odds among individuals in the >60 cups/month coffee group were lower for gastric cancer (adjusted odds ratio (aOR) = 0.80 (95% confidence intervals = 0.65–0.98)), hepatic cancer (0.32 (0.18–0.58)), colon cancer (0.53 (0.39–0.72)), breast cancer (0.56 (0.45–0.70)), and thyroid cancer (0.71 (0.59–0.85)) than for individuals in the no coffee group. Physical exercise of ≥150 min/week was correlated with higher odds for gastric cancer (1.18 (1.03–1.36)), colon cancer (1.52 (1.26–1.83)), breast cancer (1.53 (1.35–1.74)), thyroid cancer (1.42 (1.27–1.59)), and prostate cancer (1.61 (1.13–2.28)) compared to no exercise. Coffee consumption and physical exercise showed an interaction in thyroid cancer (*p* = 0.002). Coffee consumption was related to a decreased risk of gastric cancer, hepatic cancer, colon cancer, breast cancer, and thyroid cancer in the adult population. Physical exercise was positively correlated with gastric cancer, colon cancer, breast cancer, thyroid cancer, and prostate cancer.

## 1. Introduction

Coffee is consumed by people worldwide. The amount of coffee consumed was estimated to be approximately 4.1 kg/person/year in Europe and as high as approximately 12 kg/person/year in Finland [1]. About 87.30% of worldwide population including Korean consumed one or more cup of coffee/day [2]. Coffee contains more than 2000 types of compounds, including a number of bioactive substances called polyphenols, such as caffeine, caffeic acid, and chlorogenic acids [3]. These bioactive components have been reported to have antioxidative, anti-inflammatory, and antiangiogenic effects in in vitro and in vivo studies [1,3]. Thus, many previous studies have suggested the impacts of coffee consumption on health outcomes [4]. A meta-analysis demonstrated a 17% decreased risk of overall mortality (95% confidence interval (95% CI) = 0.83–0.88) and a 19% decreased risk of cardiovascular mortality (95% CI = 0.72–0.90) related to coffee consumption compared to no coffee consumption [4].

A number of previous studies have reported the effects of coffee consumption on the risk of cancers [5,6,7]. Some researchers found no effects or adverse effects of coffee consumption on the risk of some types of cancers [5,8]. In a case–control study, the relative risk for renal cell carcinoma was 4.5 times higher in women who consumed coffee [8]. A meta-analysis demonstrated no relation of coffee consumption with overall cancer risk [5]. However, a number of epidemiological studies have demonstrated a differential association of coffee consumption with cancer risk according to the type of cancer [5]. Favorable effects of coffee consumption were reported for liver cancer (relative risk (RR) = 0.92, 95% CI = 0.88–0.96) and endometrial cancer (RR = 0.92, 95% CI = 0.88–0.96) [5]. In addition, a cancer risk reduction associated with coffee consumption was identified for biliary tract cancer (RR = 0.85, 95% CI = 0.82–0.88) in a meta-analysis of prospective cohort studies in United States of America, Japan, Sweden, China, Finland, and Italy [6], and colon cancer (RR = 0.91, 95% CI = 0.83–0.998) in a meta-analysis of prospective studies in Sweden, USA, Japan, Finland, Netherlands, EPIC study, Singapore, and Norway [7]. However, because of the diverse study designs and study participant ethnicities in previous studies, it has been difficult to compare the association of coffee consumption with the risk of various types of cancers and understudies in Korea.

In addition to coffee consumption, physical exercise has been suggested as a protective factor for the risk of cancer in a review analysis [9,10,11]. Coffee consumption may have energizing effects that can promote physical exercise and attenuate fatigue [12,13]. The increased physical exercise has been suggested as protective factors for cancer-related fatigue, which might be a risk factor of reduced survival [14,15]. These alertness effects of coffee may be linked with the potential anticancer effects of coffee consumption. Because coffee consumption reduces tiredness and promotes energy, a cross-sectional study demonstrated a positive association of coffee consumption with physical exercise (odds ratio (OR) = 1.17, 95% CI = 1.04–1.32) [13]. However, to the best of our knowledge, there has been little prior research on the interaction of coffee consumption with physical exercise for the impact on the risk of cancer.

We postulated that the impacts of coffee consumption on cancer risk might be different according to both the type of cancers and the amount of coffee consumed. In addition, we hypothesized that the impact of coffee consumption on the risk of cancer could be influenced by physical exercise. To test this assumption, the prevalence of various types of cancers—gastric, hepatic, colon, breast, uterine cervix, lung, thyroid, prostate, and bladder cancer—that are most common types in Korea, was investigated in participants with different levels of coffee consumption and physical exercise. In addition, the interaction between coffee consumption and physical exercise was investigated in patients with various types of cancer.

## 2. Materials and Methods

### 2.1. Study Population and Data Collection

This cross-sectional study used data from the prospective cohort study of the Korean Genome and Epidemiology Study (KoGES) from 2004 through 2016. A detailed description of these data can be found in a previous study. Among the KoGES Consortium, we used health examinees (HEXA) data comprising information on urban residents aged ≥40 years. Data were first retrieved from the 2004–2013 database (*n* = 173,202), and follow-up data were retrieved from the 2012–2016 database (*n* = 65,611). The ethics committee of Hallym University (2019-02-020) approved the use of these data.

### 2.2. Selection of Participants

**Figure 1:**
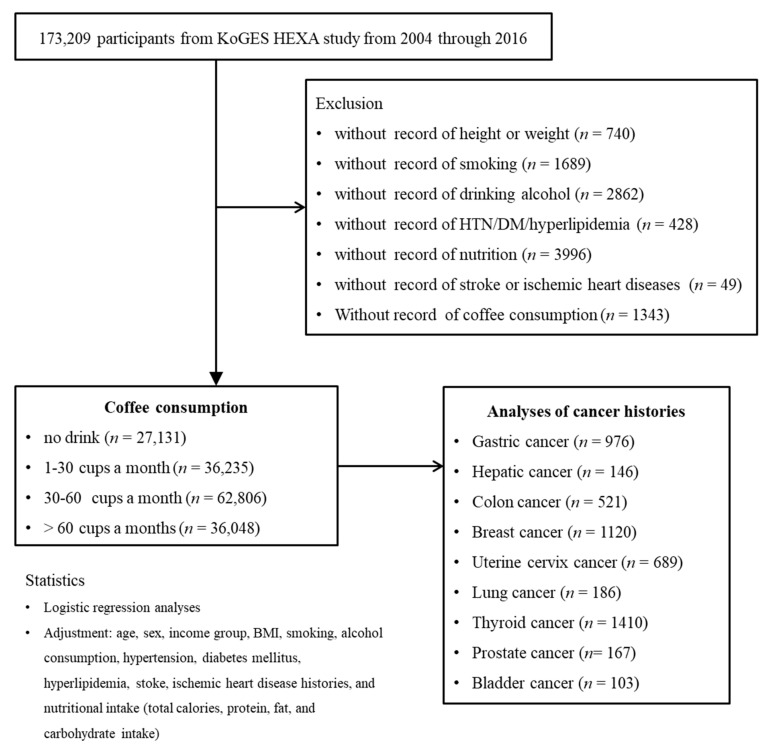
A schematic illustration of the participant selection process that was used in the present study. Of a total of 173,209 participants, 162,220 participants were involved in this study. Participants were categorized into 4 groups (no drink (*n* = 27,131): 1–30 cups a month (*n* = 36,235), 30–60 cups a month (*n* = 62,806), and >60 cups a month (*n* = 36,048)).

Among 173,209 participants, we excluded those who lacked records of height or weight (*n* = 740); smoking history (*n* = 1689); alcohol consumption (*n* = 2862); histories of hypertension, diabetes mellitus, or hyperlipidemia (*n* = 428); nutrition records (*n* = 3996); histories of ischemic heart disease or stroke (*n* = 49); or coffee consumption (*n* = 1343). The participants who lacked record of physical exercise were excluded. As a consequence, 162,220 participants were involved in this study. Participants were categorized into 4 groups (no drink (*n* = 27,131): 1–30 cups a month (*n* = 36,235), 30–60 cups a month (*n* = 62,806), and >60 cups a month (*n* = 36,048)). Then, we analyzed their histories of gastric cancer (*n* = 976), hepatic cancer (*n* = 146), colon cancer (*n* = 521), breast cancer (*n* = 1120), uterine cervix cancer (*n* = 689), lung cancer (*n* = 186), thyroid cancer (*n* = 1410), prostate cancer (*n* = 167), and bladder cancer (*n* = 103) (Figure 1).

### 2.3. Survey

#### 2.3.1. Exposure

The survey was conducted using a standardized questionnaire applied by trained interviewers [16]. Coffee consumption was assessed according to frequency and amount. Frequency was categorized as never, 1 time per month, 2–3 times per month, 1–2 times per week, 3–4 times per week, 5–6 times per week, 1–2 times per day, 3–4 times per day, and ≥5 times per day. Amount was categorized as 1/2 cup per time, 1 cup per time, and 2 cups per time. By multiplying the frequency and amount, we recategorized participants into 4 groups: none, 1–30 cups per month, 30–60 cups per month, and >60 cups per month.

Physical exercise was assessed by the survey of average excise time enough to sweat per week. The questionnaires were as follows. “How many times do you exercise enough to sweat per week? How many minutes at one time, meanly?” We categorized them as 0 (no regular exercise at all), <150 min/week, and ≥150 min/week [17].

#### 2.3.2. Outcome

The participants were asked about their histories of gastric cancer, hepatic cancer, colon cancer, breast cancer, uterine cervix cancer, lung cancer, thyroid cancer, prostate cancer, and bladder cancer by trained interviewers. They were also asked about their hypertension, diabetes mellitus, hyperlipidemia, ischemic heart disease (angina or myocardial infarction), and stroke (hemorrhagic or ischemic) histories.

#### 2.3.3. Covariates

Sex, obesity, income level, smoking status, alcohol consumption, and histories of hypertension, diabetes mellitus, hyperlipidemia, stroke, and ischemic heart disease were considered as categorical variables. Age and nutritional intake of total calories, protein, fat, and carbohydrate were considered as continuous variables. Body mass index (BMI, kg/m^2^) was measured as a part of the health checkup. Smoking history was classified as never smoker (<100 cigarettes throughout their entire life), former smoker (more than one year smoke-free), and current smoker. Alcohol consumption was classified as never drinker, former drinker, and current drinker. Nutritional intake (total calories (kcal/day), proteins (g/day), fat (g/day), and carbohydrates (g/day)) was surveyed by a food frequency questionnaire, which was validated by a previous study. Income was classified into the following groups: nonrespondent, low income (less than ~$2000 per month), middle income (~$2000–$3999 per month), and high income (~≥$4000 per month).

### 2.4. Statistics

The chi-square test was used for comparisons related to sex, obesity, income group, smoking, alcohol consumption, and histories of hypertension, diabetes mellitus, dyslipidemia, ischemic heart disease, stroke, and cancer. ANOVA was used for comparisons of age and nutritional intake.

A logistic regression model was used to analyze the odds ratio (OR) of coffee consumption and physical exercise for various types of cancer. Crude and adjusted models (adjusted for age, sex, income group, BMI, smoking, alcohol consumption, hypertension, diabetes mellitus, hyperlipidemia, stroke, ischemic heart disease histories, nutritional intake (total calories, protein, fat, and carbohydrate intake), and coffee consumption or physical exercise) were used. Except for age, and nutritional intake, other variables were managed as categorical variables. The interaction between coffee consumption and physical exercise was also calculated as creating coffee group * exercise group variables. During these calculation, coffee consumption, and exercise group were managed as the continuous variables. In the subgroup analyses according to age, the division point was set as the median age (<53 years and ≥53 years).

Two-tailed analyses were conducted, and *p* values of less than 0.05 were considered statistically significant. The results were statistically analyzed using SPSS v. 24.0 (IBM, Armonk, NY, USA).

## 3. Results

The prevalence of gastric cancer, hepatic cancer, colon cancer, breast cancer, uterine cervix cancer, lung cancer, and thyroid cancer were different according to coffee consumption history (all *p* < 0.001, Table 1). The durations of physical exercise were different according to coffee consumption history (*p* < 0.001). The coffee consumption groups showed a higher mean age, income levels, and nutritional intake of total calories, protein, fat, and carbohydrates and higher rates of male, obesity, current smoking, and current alcohol consumption (all *p* < 0.001). The histories of hypertension, diabetes mellitus, hyperlipidemia, stroke, and ischemic heart disease were lower in the coffee consumption groups (all *p* < 0.001). All variables of age, sex, obesity, income level, smoking status, alcohol consumption, histories of hypertension, diabetes mellitus, hyperlipidemia, stroke, and ischemic heart disease, nutritional intake of total calories, protein, fat, and carbohydrate, and coffee consumption were different according to the frequencies of physical exercise (Table S1). The histories of gastric cancer, colon cancer, breast cancer, thyroid cancer, and prostate cancer were higher in the frequent physical exercise groups (≥150 min/week) compared to no regular exercise group (all *p* < 0.001).

Coffee consumption was related to lower odds for gastric cancer, hepatic cancer, colon cancer, breast cancer, and thyroid cancer (Table 2). In the adjusted model, the 1–30 coffee cups/month were correlated with 0.71 (95% CI = 0.58–0.86) odds for gastric cancer, 0.54 (0.33–0.89) odds for hepatic cancer, and 0.78 (0.66–0.92) odds for breast cancer. The 30–60 coffee cups/month were related with 0.82 (0.69–0.98) odds for gastric cancer, 0.75 (0.59–0.94) odds for colon cancer. 0.72 (0.61–0.84) odds for breast cancer, 0.66 (0.45–0.98) odds for lung cancer, and 0.78 (0.67–0.90) odds for thyroid cancer in adjusted model. The >60 coffee cups/month of coffee consumption was correlated with the lower odds for gastric cancer, hepatic cancer, colon cancer, breast cancer, and thyroid cancer.

**Figure 2:**
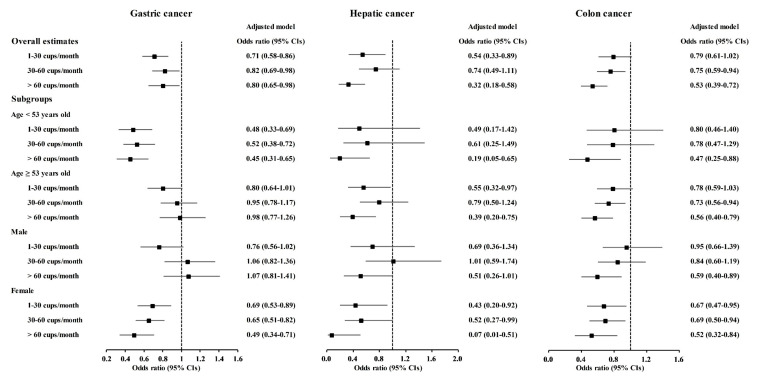
Odds ratios of coffee drinking habit for gastric cancer, hepatic cancer, and colon cancer histories according to age and sex.

**Figure 3:**
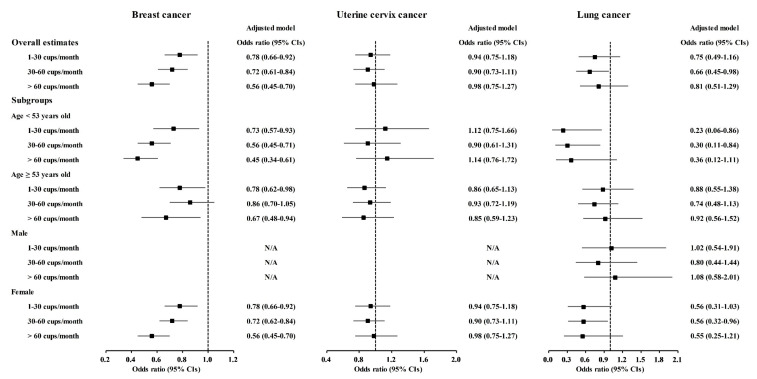
Odds ratios of coffee drinking habit for breast cancer, uterine cervix cancer, and lung cancer histories according to age and sex.

**Figure 4:**
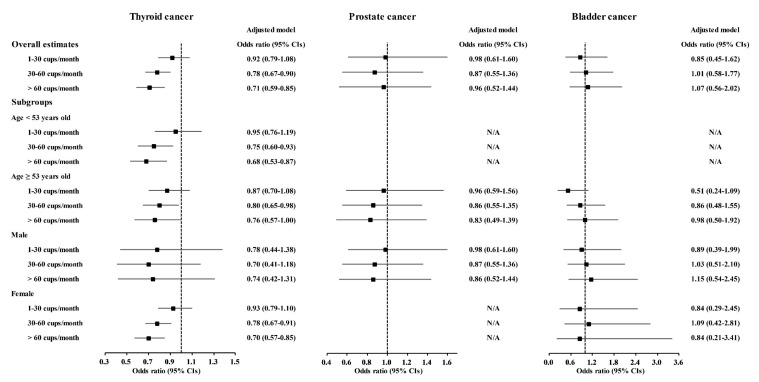
Odds ratios of coffee drinking habit for thyroid cancer, prostate cancer, and bladder cancer histories according to age and sex.

A correlation of coffee consumption with lower odds of cancer was also found in the age and sex subgroups (Figure 2 and Tables S1–S10). In the <53-year-old group, coffee consumption was correlated with lower odds for gastric cancer, hepatic cancer, colon cancer, breast cancer, lung cancer, and thyroid cancer (Figure 2 and Figure 3, Tables S2–S4, S5, S7 and S8). In the ≥53-year-old group, coffee consumption was correlated with lower odds of hepatic cancer, colon cancer, breast cancer, and thyroid cancer (Figure 2, Figure 3 and Figure 4, Tables S3–S5 and S8). In the male group, coffee consumption was correlated with lower odds of colon cancer (Figure 2, Table S3). In the female group, coffee consumption was correlated with lower odds of gastric cancer, hepatic cancer, colon cancer, breast cancer, lung cancer, and thyroid cancer (Figure 2, Figure 3 and Figure 4, Tables S2–S5, S7 and S8).

Physical exercise was correlated with higher odds for gastric cancer, colon cancer, breast cancer, thyroid cancer, and prostate cancer (Table 3). Physical exercise duration of ≥150 min/week was correlated to 1.18 (1.03–1.36) odds for gastric cancer, 1.52 (1.26–1.83) odds for colon cancer, 1.53 (1.35–1.74) odds for breast cancer, 1.42 (1.27–1.59) odds for thyroid cancer, and 1.61 (1.13–2.28) odds for prostate cancer. The <150 min/week of physical exercise was related to 1.21 (1.00–1.45) odds for breast cancer.

**Figure 5:**
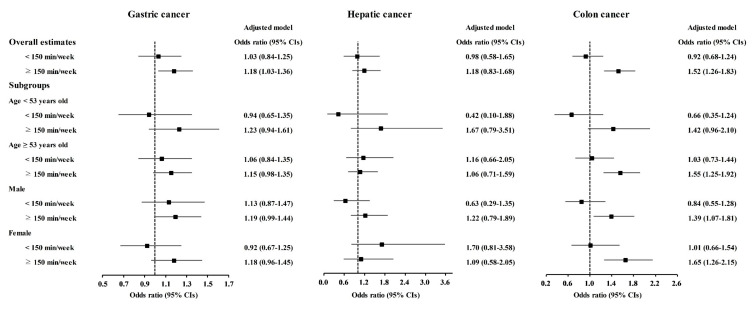
Odds ratios of physical exercise for gastric cancer, hepatic cancer, and colon cancer histories according to age and sex.

**Figure 6:**
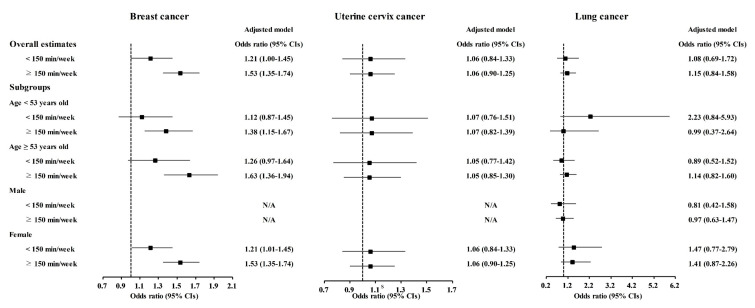
Odds ratios of physical exercise for breast cancer, uterine cervix cancer, and lung cancer histories according to age and sex.

**Figure 7:**
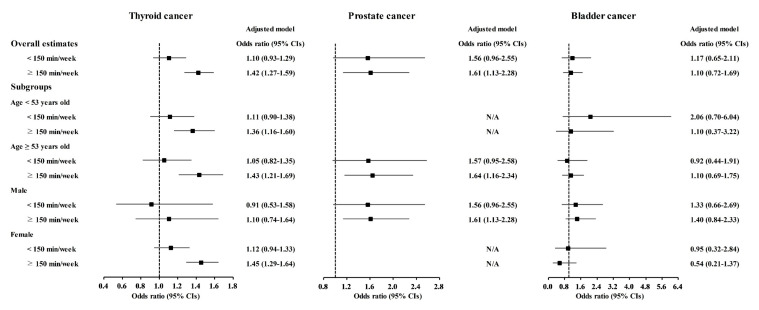
Odds ratios of physical exercise for thyroid cancer, prostate cancer, and bladder cancer histories according to age and sex.

The relationship of physical exercise with higher odds for cancers was also found in subgroup analyses according to age and sex (Figure 5, Figure 6 and Figure 7 and Tables S11–S20). The ≥150 min/week of physical exercise was correlated with higher odds for colon cancer in the ≥53 years old, male, and female subgroups, breast cancer in all age and sex subgroups, thyroid cancer in both age subgroups and female subgroups, and prostate cancer in the ≥53 years old and male subgroups.

The interaction between coffee consumption and physical exercise was significant in thyroid cancer (*p* = 0.002, Table 4 and Table S21). In subgroup analyses by coffee consumption, physical activity was positively associated with thyroid cancer in the ≥150 min/week of physical exercise group (adjusted OR (aOR) = 1.31, 95% CI = 1.03–1.68 for no drink; aOR = 1.31, 95% CI = 1.04–1.65 for 1–30 cups/month; aOR = 1.35, 95% CI = 1.01–1.82 for >60 cups/month, Table S22). The 30–60 cups/month of coffee consumption were negatively associated with thyroid cancer in no and <150 min/week of physical exercise subgroups (aOR = 0.72, 95% CI = 0.58–0.91 for no regular exercise; aOR = 0.62, 95% CI = 0.41–0.94 for <150 min/week of physical exercise, Table S23). In ≥150 min/week of physical exercise group, the >60 cups/month of coffee consumption was negatively associated with thyroid cancer (aOR = 0.71, 95% CI = 0.53–0.95)

## 4. Discussion

Coffee consumption of more than >60 cups/month and moderate or low levels of coffee consumption were correlated with a lower occurrence of gastric cancer, hepatic cancer, colon cancer, breast cancer, and thyroid cancer. Physical exercise was related to higher odds of gastric cancer, colon cancer, breast cancer, thyroid cancer, and prostate cancer. The current results filled a prior knowledge gap by demonstrating the dose-dependent and cancer-type-specific relations of coffee consumption and physical exercise with the occurrence of cancer.

There was an interaction between coffee consumption and physical exercise in patients with thyroid cancer. Physical exercise was positively associated with thyroid cancer only in high level of physical exercise group. Because this study was a cross-sectional study, the effect of cancer survivors, who pursue healthy behaviors, can be possible. The high level of physical exercise group did not show the relation of moderate coffee consumption with lower occurrence of thyroid cancer. It can be assumed that moderate coffee consumption has protective effect on thyroid cancer in subjects with high level of physical exercise. Moreover, because physical exercise was higher in patients with cancer, it can be presumed that the impact of coffee consumption on cancer was not mediated by the increased level of physical exercise. In addition to increased levels of physical exercise, coffee consumption may have a protective effect on cancer.

Several prior clinical studies suggested the cancer protective effects of coffee consumption [10,18,19]. The concordant association of coffee consumption with protection from gastric cancer, hepatic cancer, breast cancer, and thyroid cancer has been reported in both Western and Asian studies [10,18,19,20,21,22]. Our cohort also showed a negative association of coffee consumption with the occurrence of these types of cancers. The risk of gastric cancer was found to be lower among coffee consumers in a meta-analysis of case–control studies (RR = 0.85, 95% CI = 0.77–0.95) [18]. In an ecological study, countries with high coffee consumption showed a decreased incidence and mortality of gastric cancer compared to countries with lower coffee consumption (Spearman’s correlation r = −0.5984, *p* = 0.0016) [19]. The incidence of hepatic cancer also showed an inverse association with coffee consumption in a meta-analysis study in Japan (RR = 0.50, 95% CI = 0.38–0.66, *p* < 0.001) [20]. The risk of breast cancer was lower in the coffee consumption group than in the no coffee consumption group (hazard ratio = 0.44, 95% CI = 0.21–0.92) [21]. Hospital-based case–control studies described a beneficial effect of coffee consumption on thyroid cancer risk (OR = 0.59, 95% CI = 0.37–0.93) [22].

The correlation between coffee consumption and colon cancer has been controversial [23,24]. A few meta-analysis studies have reported no definite protective effect of coffee consumption on colon cancer [23,24]. In Asian cohort studies, there was no definite association between coffee consumption and colon cancer risk [23]. In addition to ethnic differences due to genetic susceptibility, differences in dietary patterns could impact the association of coffee consumption with gastrointestinal cancers [25]. The increasing adoption of Western lifestyles, especially Western dietary habits, has been proposed as a risk factor for colorectal cancers [26]. The low incidence of colorectal cancer in Asian countries compared to that in Western countries could influence the association of coffee consumption with colon cancer, although the incidence of colorectal cancer in Asian countries has recently been increasing [26]. The present results indicated the inverse association of coffee consumption with the occurrence of colon cancer. The adjustments for nutritional intake, BMI levels, smoking, and alcohol consumption may mitigate the confounding effects in the association of coffee consumption with colon cancer in the present study. Similar to the present results, a prospective cohort study reported a 12% risk reduction for overall colorectal cancer in the decaffeinated coffee consumption group compared to the caffeinated coffee consumption group (95% CI = 0.69–0.96, *p* = 0.04) [7]. In addition, a meta-analysis demonstrated an inverse association of coffee consumption with the risk of colon cancer in the overall population (RR = 0.91, 95% CI = 0.83–0.998) and some ethnic and sex subgroups of European male (RR = 0.85, 95% CI = 0.72–0.99) and Asian female (RR = 0.73, 95% CI = 0.58–0.88) [7].

The components of coffee, such as coffee polyphenols, may mediate the anticancer effects of coffee consumption [27,28,29]. A major coffee polyphenol is chlorogenic acid, which accounts for approximately 3% of roasted coffee powder [3]. Cholorogenic acid has been reported to reduce reactive oxygen species, thereby impeding inflammation and angiogenesis via molecular cascades involving NF-κB, EGFR, and VEGF [3]. Moreover, the detoxification process could be promoted by coffee consumption [30]. A randomized trial demonstrated an increase in detoxification capacity after the consumption of unfiltered coffee, as evidenced by glutathione and aminothiol levels [30]. In addition to the direct effects of coffee ingredients on cancer cells, the indirect effects of coffee consumption could exert protective effects on cancers.

Lung cancer, prostate cancer, and bladder cancer were not correlated with coffee consumption in the present study. Several studies estimated the risk of these cancers [31,32,33,34]. For lung cancer, a prospective cohort study reported an increased risk of lung cancer associated with coffee consumption (hazard ratio (HR) = 1.30, 95% CI = 1.15–1.47) [31]. Another prospective cohort study also reported a higher risk of lung cancer associated with coffee consumption (HR = 1.18, 95% CI = 1.02–1.36) [32]. Regarding prostate cancer, a meta-analysis study reported a decreased risk of prostate cancer associated with coffee consumption (pooled relative risk = 0.988, 95% CI = 0.981–0.995) [33]. For bladder cancer, a meta-analysis study did not show a significant association between the risk of bladder cancer and coffee consumption [34]. The insufficient sample size for lung cancer, prostate cancer, and bladder cancer could mitigate the potential association with coffee consumption in this study.

Physical exercise was positively correlated with gastric cancer, colon cancer, breast cancer, thyroid cancer, and prostate cancer in the present study. Previous studies mentioned the protective effects of physical exercise on cancer [11,35,36]. Physical exercise decreased the risk of colon cancer, breast cancer, and endometrial cancer [11]. Both direct impacts on tumor factors, such as suppression of tumor growth and immunologic control of tumors, and indirect impacts of whole-body exercise effects and diminished cancer-associated complications have been attributed to plausible mechanisms for the protective effects of physical activities on cancer [37,38]. The positive correlation of physical exercise with cancer in the present results might be due to health-seeking behavior in patients with cancer. Because this study was a cross-sectional study, the temporal association between physical exercise and diagnosis cancer could not be delineated. It was also possible that cancer patients who exercise are more likely to survive for a longer period in contrast to those who do not. In addition, we can suppose that the protective effects of coffee consumption on cancer were not correlated with the increased physical exercise related to high coffee consumption. Because physical exercise was higher in patients with cancer, the impact of coffee consumption on cancer might be linked with other mechanisms, including detoxification or anticancer effects of the ingredients of coffee.

This study used the large cohort of KoGES [39]. KoGES provided a large study population; thus, we could estimate the impacts of various amounts of coffee consumption on many types of cancer in this study. In addition, the possible confounders of obesity, income level, smoking, alcohol consumption, nutritional intake, and past histories of hypertension, diabetes mellitus, hyperlipidemia, stroke, and ischemic heart disease were considered when analyzing the correlation of coffee consumption with the occurrence of cancers. However, there could be other possible confounders, such as stress levels and duration or quality of sleep. Although smoking status was included, the residual confounding by tobacco smoking could influence on the association of coffee consumption and cancer, since heavy coffee drinkers tend to be smokers more frequently. Although the study population was large, the number of participants with certain types of cancers, such as hepatic cancer, lung cancer, prostate cancer, and bladder cancer, was not large, which could influence the results of the association between coffee consumption and the occurrence of cancers. For coffee consumption, the types of coffee, such as roasted, unfiltered, or decaffeinated, were heterogeneous in this study. The duration of coffee consumption, strong or week, black or with mild and sugar, standard cup sizes, espresso or black coffee, and temperature were not accessed. Moreover, because the coffee consumption and physical exercise were surveyed by self-reported questionnaires, the recall bias was possible. Last, due to the cross-sectional study design, the causality between coffee consumption or physical exercise and the occurrence of cancers could not be determined in the current study. This study was based on the self-reported cancer history; thus, the survival bias could not be excluded.

## 5. Conclusions

Coffee consumption was correlated with a lower occurrence of gastric cancer, hepatic cancer, colon cancer, breast cancer, and thyroid cancer in the adult population. Both low and high levels of coffee consumption were related to a decreased occurrence of certain types of cancer. The impacts of coffee consumption on the occurrence of cancer might be valid without interaction with elevated physical exercise.

## Figures and Tables

**Table 1:** General Characteristics of Participants.

Characteristics		Total Participants				*p* Value
		No Drink	1–30 Cups/Month	30–60 Cups/Month	>60 Cups/Month	
Number (*n*)		27,131	36,235	62,806	36,048	
Age (mean, SD, y)		55.7 (8.3)	53.7 (8.3)	53.1 (8.3)	51.0 (8.0)	<0.001 *
Sex (*n*, %)						<0.001 *
	Male	7145 (26.3)	11,092 (30.6)	19,986 (31.8)	17,387 (48.2)	
	Female	19,986 (73.7)	25,143 (69.4)	42,820 (68.2)	18,661 (51.8)	
Obesity (BMI, kg/m^2^, *n*, %)						<0.001 *
	Underweight (<18.5)	781 (2.9)	613 (1.7)	979 (1.6)	533 (1.5)	
	Normal (18.5–23)	11,487 (42.3)	13,618 (37.6)	22,993 (36.6)	12,604 (35.0)	
	Overweight (23–25)	7373 (27.2)	10,148 (28.0)	17,562 (28.0)	9985 (27.7)	
	Obese (≥ 25)	7490 (27.6)	11,856 (32.7)	21,272 (33.9)	12,926 (35.9)	
Income (*n*, %)						<0.001 *
	Missing, no response	4328 (16.0)	5439 (15.0)	7258 (11.6)	3667 (10.2)	
	Lowest	9386 (34.6)	10,539 (29.1)	17,459 (27.8)	8597 (23.8)	
	Middle	8935 (32.9)	12,965 (35.8)	24,042 (38.3)	14,591 (40.5)	
	Highest	4482 (16.5)	7292 (20.1)	14,047 (22.4)	9193 (25.5)	
Smoking status (*n*, %)						<0.001 *
	Never smoker	22,552 (83.1)	28,298 (78.1)	47,058 (74.9)	20,405 (56.6)	
	Former smoker	3142 (11.6)	4951 (13.7)	9230 (14.7)	6465 (17.9)	
	Current smoker	1437 (5.3)	2986 (8.2)	6518 (10.4)	9178 (25.5)	
Alcohol consumption (*n*, %)						<0.001 *
	Never drinker	18,248 (67.3)	19,385 (53.5)	31,008 (49.4)	13,986 (38.8)	
	Former drinker	1171 (4.3)	1472 (4.1)	2076 (3.3)	1533 (4.3)	
	Current drinker	7712 (28.4)	15,378 (42.4)	29,722 (47.3)	20,529 (56.9)	
Hypertension (*n*, %)		6946 (25.6)	8624 (23.8)	14,374 (22.9)	6604 (18.3)	<0.001 *
Diabetes mellitus (*n*, %)		2701 (10.0)	3201 (8.8)	4836 (7.7)	2231 (6.2)	<0.001 *
Hyperlipidemia (*n*, %)		4042 (14.9)	5317 (14.7)	8304 (13.2)	4023 (11.2)	<0.001 *
Stroke (*n*, %)		566 (2.1)	493 (1.4)	809 (1.3)	411 (1.1)	<0.001 *
Ischemic heart disease (*n*, %)		1077 (4.0)	1163 (3.2)	1781 (2.8)	929 (2.6)	<0.001 *
Nutritional intake (mean, SD)						
	Total calories (kcal/d)	1631.8 (545.9)	1666.3 (591.0)	1774 (539.8)	1901.9 (631.2)	<0.001 *
	Protein (g/d)	55.3 (25.5)	56.0 (27.6)	60.4 (24.7)	65.4 (29.3)	<0.001 *
	Fat (g/d)	23.1 (16.4)	25.3 (18.7)	28.6 (16.8)	33.6 (20.5)	<0.001 *
	Carbohydrate (g/d)	296.4 (91.7)	298.9 (96.4)	315.0 (90.0)	330.1 (101.7)	<0.001 *
Exercise						<0.001 *
	No regular exercise	13,541 (49.9)	17,099 (47.2)	31,241 (49.7)	20,214 (56.1)	
	<150 min/week	3698 (13.6)	6079 (16.8)	8948 (14.2)	4942 (13.7)	
	≥150 min/week	9892 (36.5)	13,057 (36.0)	22,617 (36.0)	10,892 (30.2)	
Gastric cancer		237 (0.9)	188 (0.5)	360 (0.6)	191 (0.5)	<0.001 *
Hepatic cancer		42 (0.2)	26 (0.1)	60 (0.1)	18 (0.0)	<0.001 *
Colon cancer		130 (0.5)	121 (0.3)	194 (0.3)	76 (0.2)	<0.001 *
Breast cancer ^†^		310 (1.6)	275 (1.1)	408 (1.0)	124 (0.7)	<0.001 *
Uterine cervix cancer ^†^		153 (0.8)	165 (0.7)	259 (0.6)	112 (0.6)	<0.001 *
Lung cancer		47 (0.2)	40 (0.1)	60 (0.1)	39 (0.1)	0.016 *
Thyroid cancer		301 (1.1)	363 (1.0)	527 (0.8)	219 (0.6)	<0.001 *
Prostate cancer ^†^		30 (0.4)	39 (0.4)	62 (0.3)	36 (0.2)	0.946
Bladder cancer		19 (0.1)	19 (0.1)	39 (0.1)	26 (0.1)	0.724

* ANOVA or Chi-square test. Significance at *p* < 0.05, SD: standard deviation, BMI: body mass index. ^†^ Breast and uterine cervix cancer was calculated in female, and prostate cancer was in male.

**Table 2:** Crude and adjusted odd ratios (95% confidence interval) of coffee drinking habit for various cancer histories.

Type of Cancer		*N* of Cancer	*N* of Control	OR (95% CI)	
		(Exposure/Total, %)	(Exposure/Total, %)	Crude	Adjusted ^†^
Gastric cancer					
	No drink	237/976 (24.3%)	26,894/161,244 (16.7%)	1.00	1.00
	1–30 cups/month	188/976 (19.3%)	36,047/161,244 (22.4%)	0.59 (0.49–0.72) *	0.71 (0.58–0.86) *
	30–60 cups/month	360/976 (36.9%)	62,446/161,244 (38.7%)	0.65 (0.56–0.77) *	0.82 (0.69–0.98) *
	>60 cups/month	191/976 (19.6%)	35,857/161,244 (22.2%)	0.60 (0.50–0.73) *	0.80 (0.65–0.98) *
Hepatic cancer					
	No drink	42/146 (27.8%)	27,089/162,074 (16.7%)	1.00	1.00
	1–30 cups/month	26/146 (17.8%)	36,209/162,074 (22.3%)	0.46 (0.28–0.76) *	0.54 (0.33–0.89) *
	30–60 cups/month	60/146 (41.1%)	62,746/162,074 (38.7%)	0.62 (0.42–0.92) *	0.74 (0.49–1.11)
	>60 cups/month	18/146 (12.3%)	36,030/162,074 (22.2%)	0.32 (0.19–0.56) *	0.32 (0.18–0.58) *
Colon cancer					
	No drink	130/521 (24.9%)	27,001/161,699 (16.7%)	1.00	1.00
	1–30 cups/month	121/521 (23.2%)	36,114/161,699 (22.3%)	0.70 (0.54–0.89) *	0.79 (0.61–1.02)
	30–60 cups/month	194/521 (37.2%)	62,612/161,699 (38.7%)	0.64 (0.52–0.80) *	0.75 (0.59–0.94) *
	>60 cups/month	76/521 (14.6%)	35,972/161,699 (22.2%)	0.44 (0.33–0.58) *	0.53 (0.39–0.72) *
Breast cancer ^‡^					
	No drink	310/1117 (27.8%)	19,676/105,493 (18.7%)	1.00	1.00
	1–30 cups/month	275/1117 (24.6%)	24,868/105,493 (23.6%)	0.70 (0.60–0.83) *	0.78 (0.66–0.92) *
	30–60 cups/month	408/1117 (36.5%)	42,412/105,493 (40.2%)	0.61 (0.53–0.71) *	0.72 (0.61–0.84) *
	>60 cups/month	124/1117 (11.1%)	18,537/105,493 (17.6%)	0.43 (0.34–0.52) *	0.56 (0.45–0.70) *
Uterine cervix cancer ^‡^					
	No drink	153/689 (22.2%)	19,833/105,921 (18.7%)	1.00	1.00
	1–30 cups/month	165/689 (23.9%)	24,978/105,921 (23.6%)	0.86 (0.69–1.07)	0.94 (0.75–1.18)
	30–60 cups/month	259/689 (37.6%)	42,561/105,921 (40.2%)	0.79 (0.65–0.96) *	0.90 (0.73–1.11)
	>60 cups/month	112/689 (16.3%)	18,549/105,921 (17.5%)	0.78 (0.61–1.00) *	0.98 (0.75–1.27)
Lung cancer					
	No drink	47/186 (25.3%)	27,084/162,034 (16.7%)	1.00	1.00
	1–30 cups/month	40/186 (21.5%)	36,195/162,034 (22.3%)	0.64 (0.42–0.97) *	0.75 (0.49–1.16)
	30–60 cups/month	60/186 (32.3%)	62,746/162,034 (38.7%)	0.55 (0.38–0.81) *	0.66 (0.45–0.98) *
	>60 cups/month	39/186 (21.0%)	36,009/162,034 (22.2%)	0.62 (0.41–0.95) *	0.81 (0.51–1.29)
Thyroid cancer					
	No drink	301/1410 (21.3%)	26,830/160,810 (16.7%)	1.00	1.00
	1–30 cups/month	363/1410 (25.7%)	35,872/160,810 (22.3%)	0.90 (0.77–1.05)	0.92 (0.79–1.08)
	30–60 cups/month	527/1410 (37.4%)	62,279/160,810 (38.7%)	0.75 (0.65–0.87) *	0.78 (0.67–0.90) *
	>60 cups/month	219/1410 (15.5%)	35,829/160,810 (22.3%)	0.55 (0.46–0.65) *	0.71 (0.59–0.85) *
Prostate cancer ^‡^					
	No drink	30/167 (18.0%)	7115/55,443 (12.8%)	1.00	1.00
	1–30 cups/month	39/167 (23.3%)	11,053/55,443 (19.9%)	0.84 (0.52–1.35)	0.98 (0.61–1.60)
	30–60 cups/month	62/167 (37.1%)	19,924/55,443 (35.9%)	0.74 (0.48–1.14)	0.87 (0.55–1.36)
	>60 cups/month	36/167 (21.6%)	17,351/55,443 (31.3%)	0.49 (0.30–0.80) *	0.86 (0.52–1.44)
Bladder cancer					
	No drink	19/103 (18.4%)	27,112/162,117 (16.7%)	1.00	1.00
	1–30 cups/month	19/103 (18.4%)	36,216/162,117 (22.3%)	0.75 (0.40–1.41)	0.85 (0.45–1.62)
	30–60 cups/month	39/103 (37.9%)	62,767/162,117 (38.7%)	0.89 (0.51–1.53)	1.01 (0.58–1.77)
	>60 cups/month	26/103 (25.2%)	36,022/162,117 (22.2%)	1.03 (0.57–1.86)	1.07 (0.56–2.02)

* Logistic regression model, Significance at *p* < 0.05. ^†^ The model was adjusted for age, sex, income group, body mass index, smoking, alcohol consumption, hypertension, diabetes mellitus, hyperlipidemia, stoke, ischemic heart disease histories, nutritional intake (total calories, protein, fat, and carbohydrate intake), and physical exercise. ^‡^ Breast and uterine cervix cancer was calculated in female, and prostate cancer was in male. OR: odds ratio, 95% CI: 95% confidence intervals.

**Table 3:** Crude and adjusted odd ratios (95% confidence interval) of physical exercise for various cancer histories.

Type of Cancer		*N* of Cancer	*N* of Control	OR (95% CI)	
		(Exposure/Total, %)	(Exposure/Total, %)	Crude	Adjusted ^†^
Gastric cancer					
	No regular exercise	444/976 (45.5%)	81,651/161,244 (50.6%)	1.00	1.00
	<150 min/week	128/976 (13.1%)	23,539/161,244 (14.6%)	1.00 (0.82–1.22)	1.03 (0.84–1.25)
	≥150 min/week	404/976 (41.4%)	56,054/161,244 (34.8%)	1.33 (1.16–1.52) *	1.18 (1.03–1.36) *
Hepatic cancer					
	No regular exercise	66/146 (45.2%)	82,029/162,074 (50.6%)	1.00	1.00
	<150 min/week	18/146 (12.3%)	23,649/162,074 (14.6%)	0.95 (0.56–1.59)	0.98 (0.58–1.65)
	≥150 min/week	62/146 (42.5%)	56,396/162,074 (34.8%)	1.37 (0.97–1.93)	1.18 (0.83–1.68)
Colon cancer					
	No regular exercise	211/521 (40.5%)	81,884/161,699 (50.6%)	1.00	1.00
	<150 min/week	55/521 (10.6%)	23,612/161,699 (14.6%)	0.90 (0.67–1.22)	0.92 (0.68–1.24)
	≥150 min/week	255/521 (48.9%)	56,203/161,699 (34.8%)	1.76 (1.47–2.11) *	1.52 (1.26–1.83) *
Breast cancer ^‡^					
	No regular exercise	476/1120 (42.5%)	81,619/161,100 (50.7%)	1.00	1.00
	<150 min/week	155/1120 (13.8%)	23,512/161,100 (14.6%)	1.13 (0.94–1.36)	1.21 (1.00–1.45) *
	≥150 min/week	489/1120 (43.7%)	55,969/161,100 (34.7%)	1.50 (1.32–1.70) *	1.53 (1.35–1.74) *
Uterine cervix cancer ^‡^					
	No regular exercise	355/689 (51.5%)	81,740/161,531 (50.6%)	1.00	1.00
	<150 min/week	94/689 (13.6%)	23,573/161,531 (14.6%)	0.92 (0.73–1.15)	1.06 (0.84–1.33)
	≥150 min/week	240/689 (34.8%)	56,218/161,531 (34.8%)	0.98 (0.83–1.16)	1.06 (0.90–1.25)
Lung cancer					
	No regular exercise	83/186 (44.6%)	82,012/162,034 (50.6%)	1.00	1.00
	<150 min/week	24/186 (12.9%)	23,646/162,034 (14.6%)	1.00 (0.64–1.58)	1.08 (0.69–1.72)
	≥150 min/week	79/186 (42.5%)	53,679/162,034 (34.8%)	1.38 (1.02–1.88) *	1.15 (0.84–1.58)
Thyroid cancer					
	No regular exercise	616/1410 (43.7%)	81,479/160,810 (50.7%)	1.00	1.00
	<150 min/week	191/1410 (13.5%)	23,476/160,810 (14.6%)	1.08 (0.91–1.27)	1.10 (0.93–1.29)
	≥150 min/week	603/1410 (42.8%)	55,855/160,810 (34.7%)	1.43 (1.28–1.60) *	1.42 (1.27–1.59) *
Prostate cancer ^‡^					
	No regular exercise	53/167 (31.7%)	82,042/162,053 (50.6%)	1.00	1.00
	<150 min/week	24/167 (14.4%)	23,643/162,053 (14.6%)	1.57 (0.97–2.55)	1.56 (0.96–2.55)
	≥150 min/week	90/167 (53.9%)	56,368/162,053 (34.8%)	2.47 (1.76–3.47) *	1.61 (1.13–2.28) *
Bladder cancer					
	No regular exercise	46/103 (44.7%)	82,049/162,117 (50.6%)	1.00	1.00
	<150 min/week	15/103 (14.6%)	23,652/162,117 (14.6%)	1.13 (0.63–2.03)	1.17 (0.65–2.11)
	≥150 min/week	42/103 (40.8%)	56,416/162,117 (34.8%)	1.33 (0.87–2.02)	1.10 (0.72–1.69)

* Logistic regression model, Significance at *p* < 0.05. ^†^ The model was adjusted for age, sex, income group, body mass index, smoking, alcohol consumption, hypertension, diabetes mellitus, hyperlipidemia, stoke, ischemic heart disease histories, nutritional intake (total calories, protein, fat, and carbohydrate intake), and coffee consumption. ^‡^ Breast and uterine cervix cancer was calculated in female, and prostate cancer was in male. OR: odds ratio, 95% CI: 95% confidence intervals.

**Table 4:** The interaction between coffee consumption, and physical exercise for various cancer histories.

Type of Cancer	Interaction	
	Crude (*p* Value)	Adjusted ^†^ (*p* Value)
Gastric cancer	0.298	0.230
Hepatic cancer	0.215	0.125
Colon cancer	0.190	0.380
Breast cancer ^‡^	0.946	0.370
Uterine cervix cancer ^‡^	0.937	0.366
Lung cancer	0.791	0.840
Thyroid cancer	0.113	0.002 *
Prostate cancer ^‡^	0.032 *	0.102
Bladder cancer	0.046 *	0.185

* Logistic regression model, Significance at *p* < 0.05. ^†^ The model was adjusted for age, sex, income group, body mass index, smoking, alcohol consumption, hypertension, diabetes mellitus, hyperlipidemia, stoke, ischemic heart disease histories, and nutritional intake (total calories, protein, fat, and carbohydrate intake) ^‡^ Breast and uterine cervix cancer was calculated in female, and prostate cancer was in male.

## Data Availability

Releasing of the data by the researcher is not legally permitted. All data are available from the database of the Korea Center for Disease Control and Prevention. The Korea Center for Disease Control and Prevention allows data access, at a particular cost, for any researcher who promises to follow the research ethics. The data of this article can be downloaded from the website after agreeing to follow the research ethics.

## Supplementary Materials

The following are available online at https://www.mdpi.com/article/10.3390/nu13113927/s1, Table S1: General Characteristics of Participants, Table S2: Crude and adjusted odd ratios (95% confidence interval) of coffee drinking habit for gastric cancer histories, Table S3: Crude and adjusted odd ratios (95% confidence interval) of coffee drinking habit for hepatic cancer histories, Table S4: Crude and adjusted odd ratios (95% confidence interval) of coffee drinking habit for colon cancer histories, Table S5: Crude and adjusted odd ratios (95% confidence interval) of coffee drinking habit for breast cancer histories, Table S6: Crude and adjusted odd ratios (95% confidence interval) of coffee drinking habit for uterine cervix cancer histories, Table S7: Crude and adjusted odd ratios (95% confidence interval) of coffee drinking habit for lung cancer histories, Table S8: Crude and adjusted odd ratios (95% confidence interval) of coffee drinking habit for thyroid cancer histories, Table S9 Crude and adjusted odd ratios (95% confidence interval) of coffee drinking habit for prostate cancer histories, Table S10: Crude and adjusted odd ratios (95% confidence interval) of coffee drinking habit for bladder cancer histories, Table S11: Crude and adjusted odd ratios (95% confidence interval) of physical exercise for gastric cancer histories, Table S12: Crude and adjusted odd ratios (95% confidence interval) of physical exercise for hepatic cancer histories, Table S13 Crude and adjusted odd ratios (95% confidence interval) of physical exercise for colon cancer histories, Table S14: Crude and adjusted odd ratios (95% confidence interval) of physical exercise for breast cancer histories, Table S15: Crude and adjusted odd ratios (95% confidence interval) of physical exercise for uterine cervix cancer histories, Table S16: Crude and adjusted odd ratios (95% confidence interval) of physical exercise for lung cancer histories, Table S17: Crude and adjusted odd ratios (95% confidence interval) of physical exercise for thyroid cancer histories, Table S18: Crude and adjusted odd ratios (95% confidence interval) of physical exercise for prostate cancer histories, Table S19: Crude and adjusted odd ratios (95% confidence interval) of physical exercise for bladder cancer histories, Table S20: Crude and adjusted odd ratios (95% confidence interval) of physical exercise for thyroid cancer histories, Table S21: The interaction between coffee consumption, and physical exercise for various cancer histories, Table S22: The association between physical exercise and thyroid cancer in the subgroup analyses by coffee consumption, Table S23: The association between coffee consumption and thyroid cancer in the subgroup analyses by physical exercise.
