# Supplementary material for: Association between Coffee Consumption/Physical Exercise and Gastric, Hepatic, Colon, Breast, Uterine Cervix, Lung, Thyroid, Prostate, and Bladder Cancer
Source: Nutrients. 2021 Nov 2;13(11):3927. doi: 10.3390/nu13113927 (PMC8620757; doi:10.3390/nu13113927)
Supplement: Supplementary file 1 [file nutrients-13-03927-s001.zip › nutrients-1385831-supplementary.pdf]

Table S1. General Characteristics of Participants

Characteristics	Total participants			P-value
	No regular exercise	< 150 min/week	≥ 150 min/week	
Number (n)	82,095	23,667	56,458	
Age (mean, SD, y)	52.9 (8.6)	52.1 (8.0)	54.1 (8.1)	<0.001*
Sex (n, %)				<0.001*
Male	25,953 (31.6)	9,081 (38.4)	20,576 (36.4)	
Female	56,142 (68.4)	14,586 (61.6)	35,882 (63.6)	
Obesity (BMI, kg/m^2, n, %)				<0.001*
Underweight (<18.5)	1,845 (2.2)	368 (1.6)	693 (1.2)	
Normal (18.5-23)	31,174 (38.0)	8,962 (37.9)	20,566 (36.4)	
Overweight (23-25)	21,713 (26.4)	6,771 (28.6)	16,584 (29.4)	
Obese (≥ 25)	27,363 (33.3)	7,566 (32.0)	18,615 (33.0)	
Income (n, %)				<0.001*
Missing, no response	11,315 (13.8)	2,758 (11.7)	6,619 (11.7)	
Lowest	25,933 (31.6)	5,701 (24.1)	14,347 (25.4)	
Middle	29,644 (36.1)	9,016 (38.1)	21,873 (38.7)	
Highest	15,203 (18.5)	6,192 (26.2)	13,619 (24.1)	
Smoking status (n, %)				<0.001*
Nonsmoker	60,619 (73.8)	16,910 (71.4)	40,784 (72.2)	
Past smoker	9,841 (12.0)	3,756 (15.9)	10,191 (18.1)	
Current smoker	11,635 (14.2)	3,001 (12.7)	5,483 (9.7)	
Alcohol consumption (n, %)				<0.001*
Non drinker	43,942 (53.5)	11,005 (46.5)	27,680 (49.0)	
Past drinker	2,980 (3.6)	990 (4.2)	2,282 (4.0)	
Current drinker	35,173 (42.8)	11,672 (49.3)	26,496 (46.9)	
Hypertension (n, %)	17,488 (21.3)	4,939 (20.9)	14,121 (25.0)	<0.001*
Diabetes mellitus (n, %)	5,874 (7.2)	1,700 (7.2)	5,395 (7.9)	<0.001*
Hyperlipidemia (n, %)	9,868 (12.0)	3,306 (14.0)	8,512 (15.1)	<0.001*
Stroke (n, %)	1,132 (1.4)	269 (1.1)	878 (1.6)	<0.001*
Ischemic heart disease (n, %)	2,426 (3.0)	627 (2.6)	1,897 (3.4)	<0.001*
Nutritional intake (mean, SD)				
Total calories (kcal/d)	1731.4 (579.7)	1771.4 (579.4)	1781.5 (584.3)	<0.001*
Protein (g/d)	58.3 (26.5)	60.2 (26.8)	61.4 (27.1)	<0.001*
Fat (g/d)	27.5 (18.5)	28.4 (18.5)	28.7 (18.2)	<0.001*
Carbohydrate (g/d)	308.3 (94.6)	314.4 (94.9)	315.7 (96.2)	<0.001*
Coffee consumption (n, %)				<0.001*
No drink	13,541 (16.5)	3,698 (15.6)	9,892 (17.5)	

1-30 cups/month	17,099 (20.8)	6,079 (25.7)	13,057 (23.1)	
30-60 cups/month	31,241 (38.1)	8,948 (37.8)	22,617 (40.1)	
>60 cups/month	20,214 (24.6)	4,942 (20.9)	10,892 (19.3)	
Gastric cancer	444 (0.5)	128 (0.5)	404 (0.7)	<0.001*
Hepatic cancer	66 (0.1)	18 (0.1)	62 (0.1)	0.148
Colon cancer	211 (0.3)	55 (0.2)	255 (0.5)	<0.001*
Breast cancer †	475 (0.8)	155 (1.1)	487 (1.4)	<0.001*
Uterine cervix cancer †	355 (0.6)	94 (0.6)	240 (0.7)	0.796
Lung cancer	83 (0.1)	24 (0.1)	79 (0.1)	0.089
Thyroid cancer	616 (0.8)	191 (0.8)	603 (1.1)	<0.001*
Prostate cancer †	53 (0.2)	24 (0.3)	90 (0.4)	<0.001*
Bladder cancer	46 (0.1)	15 (0.1)	42 (0.1)	0.411

* ANOVA or Chi-square test. Significance at $P < 0.05$

† Breast and uterine cervix cancer was calculated in Female, and prostate cancer was in Male.

Table S2. Crude and adjusted odd ratios (95% confidence interval) of coffee drinking habit for gastric cancer histories

Type of cancer	OR (95% CI) for gastric cancer			
	Crude	P-value	Adjusted†	P-value
Age < 53 years old				
No drink	1.00		1.00	
1-30 cups/month	0.42 (0.29-0.60)	<0.001*	0.48 (0.33-0.69)	<0.001*
30-60 cups/month	0.43 (0.31-0.58)	<0.001*	0.52 (0.38-0.72)	<0.001*
> 60 cups/month	0.36 (0.25-0.51)	<0.001*	0.45 (0.31-0.65)	<0.001*
Age ≥ 53 years old				
No drink	1.00		1.00	
1-30 cups/month	0.74 (0.59-0.92)	0.008*	0.80 (0.64-1.01)	0.058
30-60 cups/month	0.86 (0.71-1.05)	0.142	0.95 (0.78-1.17)	0.640
> 60 cups/month	0.98 (0.78-1.24)	0.893	0.98 (0.77-1.26)	0.891
Male				
No drink	1.00		1.00	
1-30 cups/month	0.59 (0.44-0.79)	<0.001*	0.76 (0.56-1.02)	0.063
30-60 cups/month	0.79 (0.62-1.00)	0.053	1.06 (0.82-1.36)	0.649
> 60 cups/month	0.63 (0.49-0.82)	<0.001*	1.07 (0.81-1.41)	0.631
Female				
No drink	1.00		1.00	
1-30 cups/month	0.56 (0.44-0.73)	<0.001*	0.69 (0.53-0.89)	0.005*
30-60 cups/month	0.49 (0.39-0.62)	<0.001*	0.65 (0.51-0.82)	<0.001*
> 60 cups/month	0.33 (0.23-0.46)	<0.001*	0.49 (0.34-0.71)	<0.001*

* Logistic regression model, Significance at P < 0.05

† The model was adjusted for age, sex, income group, BMI, smoking, alcohol consumption, hypertension, diabetes mellitus, hyperlipidemia, stoke, ischemic heart disease histories, nutritional intake (total calories, protein, fat, and carbohydrate intake), and physical exercise.

Table S3. Crude and adjusted odd ratios (95% confidence interval) of coffee drinking habit for hepatic cancer histories

Type of cancer	OR (95% CI) for hepatic cancer			
	Crude	P-value	Adjusted†	P-value
Age < 53 years old				
No drink	1.00		1.00	
1-30 cups/month	0.44 (0.15-1.27)	0.128	0.49 (0.17-1.42)	0.186
30-60 cups/month	0.52 (0.22-1.26)	0.148	0.61 (0.25-1.49)	0.279
> 60 cups/month	0.23 (0.07-0.76)	0.016*	0.19 (0.05-0.65)	0.009*
Age ≥ 53 years old				
No drink	1.00		1.00	
1-30 cups/month	0.53 (0.30-0.91)	0.022*	0.55 (0.32-0.97)	0.037*
30-60 cups/month	0.75 (0.48-1.16)	0.198	0.79 (0.50-1.24)	0.301
> 60 cups/month	0.50 (0.27-0.93)	0.028*	0.39 (0.20-0.75)	0.005*
Male				
No drink	1.00		1.00	
1-30 cups/month	0.49 (0.26-0.94)	0.032*	0.69 (0.36-1.34)	0.279
30-60 cups/month	0.70 (0.41-1.18)	0.180	1.01 (0.59-1.74)	0.975
> 60 cups/month	0.33 (0.18-0.63)	0.001*	0.51 (0.26-1.01)	0.053
Female				
No drink	1.00		1.00	
1-30 cups/month	0.38 (0.18-0.80)	0.011*	0.43 (0.20-0.92)	0.029*
30-60 cups/month	0.42 (0.23-0.79)	0.006*	0.52 (0.27-0.99)	0.047*
> 60 cups/month	0.05 (0.01-0.38)	0.004*	0.07 (0.01-0.51)	0.009*

* Logistic regression model, Significance at P < 0.05

† The model was adjusted for age, sex, income group, BMI, smoking, alcohol consumption, hypertension, diabetes mellitus, hyperlipidemia, stoke, ischemic heart disease histories, nutritional intake (total calories, protein, fat, and carbohydrate intake), and physical exercise.

Table S4. Crude and adjusted odd ratios (95% confidence interval) of coffee drinking habit for colon cancer histories

Type of cancer	OR (95% CI) for colon cancer			
	Crude	P-value	Adjusted†	P-value
Age < 53 years old				
No drink	1.00		1.00	
1-30 cups/month	0.71 (0.41-1.24)	0.231	0.80 (0.46-1.40)	0.441
30-60 cups/month	0.64 (0.39-1.06)	0.083	0.78 (0.47-1.29)	0.330
> 60 cups/month	0.40 (0.22-0.72)	0.003*	0.47 (0.25-0.88)	0.019*
Age ≥ 53 years old				
No drink	1.00		1.00	
1-30 cups/month	0.78 (0.59-1.03)	0.074	0.78 (0.59-1.03)	0.081
30-60 cups/month	0.75 (0.58-0.96)	0.022*	0.73 (0.56-0.94)	0.016*
> 60 cups/month	0.63 (0.46-0.87)	0.005*	0.56 (0.40-0.79)	0.001*
Male				
No drink	1.00		1.00	
1-30 cups/month	0.82 (0.57-1.18)	0.290	0.95 (0.66-1.39)	0.805
30-60 cups/month	0.73 (0.52-1.02)	0.064	0.84 (0.60-1.19)	0.328
> 60 cups/month	0.43 (0.29-0.63)	<0.001*	0.59 (0.40-0.89)	0.012*
Female				
No drink	1.00		1.00	
1-30 cups/month	0.56 (0.40-0.79)	0.001*	0.67 (0.47-0.95)	0.025*
30-60 cups/month	0.53 (0.39-0.72)	<0.001*	0.69 (0.50-0.94)	0.019*
> 60 cups/month	0.31 (0.20-0.50)	<0.001*	0.52 (0.32-0.84)	0.008*

* Logistic regression model, Significance at P < 0.05

† The model was adjusted for age, sex, income group, BMI, smoking, alcohol consumption, hypertension, diabetes mellitus, hyperlipidemia, stoke, ischemic heart disease histories, nutritional intake (total calories, protein, fat, and carbohydrate intake), and physical exercise.

Table S5. Crude and adjusted odd ratios (95% confidence interval) of coffee drinking habit for breast cancer histories

Type of cancer	OR (95% CI) for breast cancer			
	Crude	P-value	Adjusted†	P-value
Age < 53 years old				
No drink	1.00		1.00	
1-30 cups/month	0.63 (0.49-0.80)	<0.001*	0.73 (0.57-0.93)	0.011*
30-60 cups/month	0.46 (0.36-0.57)	<0.001*	0.56 (0.45-0.71)	<0.001*
> 60 cups/month	0.34 (0.25-0.45)	<0.001*	0.45 (0.34-0.61)	<0.001*
Age ≥ 53 years old				
No drink	1.00		1.00	
1-30 cups/month	0.75 (0.60-0.93)	0.011*	0.78 (0.62-0.98)	0.031*
30-60 cups/month	0.78 (0.64-0.95)	0.013*	0.86 (0.70-1.05)	0.135
> 60 cups/month	0.55 (0.40-0.76)	<0.001*	0.67 (0.48-0.94)	0.019*
Male				
No drink	1.00		1.00	
1-30 cups/month	N/A		N/A	
30-60 cups/month	N/A		N/A	
> 60 cups/month	N/A		N/A	
Female				
No drink	1.00		1.00	
1-30 cups/month	0.70 (0.60-0.83)	<0.001*	0.78 (0.66-0.92)	0.003*
30-60 cups/month	0.61 (0.53-0.71)	<0.001*	0.72 (0.62-0.84)	<0.001*
> 60 cups/month	0.43 (0.34-0.52)	<0.001*	0.56 (0.45-0.70)	<0.001*

* Logistic regression model, Significance at P < 0.05

† The model was adjusted for age, sex, income group, BMI, smoking, alcohol consumption, hypertension, diabetes mellitus, hyperlipidemia, stoke, ischemic heart disease histories, nutritional intake (total calories, protein, fat, and carbohydrate intake), and physical exercise.

Table S6. Crude and adjusted odd ratios (95% confidence interval) of coffee drinking habit for uterine cervix cancer histories

Type of cancer	OR (95% CI) for uterine cervix cancer			
	Crude	P-value	Adjusted†	P-value
Age < 53 years old				
No drink	1.00		1.00	
1-30 cups/month	1.06 (0.72-1.57)	0.767	1.12 (0.75-1.66)	0.586
30-60 cups/month	0.82 (0.57-1.19)	0.290	0.90 (0.61-1.31)	0.565
> 60 cups/month	1.01 (0.68-1.50)	0.950	1.14 (0.76-1.72)	0.520
Age ≥ 53 years old				
No drink	1.00		1.00	
1-30 cups/month	0.83 (0.63-1.08)	0.168	0.86 (0.65-1.13)	0.278
30-60 cups/month	0.89 (0.70-1.13)	0.321	0.93 (0.72-1.19)	0.543
> 60 cups/month	0.80 (0.57-1.14)	0.215	0.85 (0.59-1.23)	0.390
Male				
No drink	1.00		1.00	
1-30 cups/month	N/A		N/A	
30-60 cups/month	N/A		N/A	
> 60 cups/month	N/A		N/A	
Female				
No drink	1.00		1.00	
1-30 cups/month	0.86 (0.69-1.07)	0.168	0.94 (0.75-1.18)	0.609
30-60 cups/month	0.79 (0.65-0.96)	0.020*	0.90 (0.73-1.11)	0.335
> 60 cups/month	0.78 (0.61-1.00)	0.050	0.98 (0.75-1.27)	0.868

* Logistic regression model, Significance at P < 0.05

† The model was adjusted for age, sex, income group, BMI, smoking, alcohol consumption, hypertension, diabetes mellitus, hyperlipidemia, stoke, ischemic heart disease histories, nutritional intake (total calories, protein, fat, and carbohydrate intake), and physical exercise.

Table S7. Crude and adjusted odd ratios (95% confidence interval) of coffee drinking habit for lung cancer histories

Type of cancer	OR (95% CI) for lung cancer			
	Crude	P-value	Adjusted†	P-value
Age < 53 years old				
No drink	1.00		1.00	
1-30 cups/month	0.22 (0.06-0.83)	0.025*	0.23 (0.06-0.86)	0.029*
30-60 cups/month	0.28 (0.10-0.78)	0.014*	0.30 (0.11-0.84)	0.022*
> 60 cups/month	0.34 (0.12-0.99)	0.048*	0.36 (0.12-1.11)	0.074
Age ≥ 53 years old				
No drink	1.00		1.00	
1-30 cups/month	0.85 (0.54-1.33)	0.471	0.88 (0.55-1.38)	0.567
30-60 cups/month	0.74 (0.49-1.11)	0.145	0.74 (0.48-1.13)	0.158
> 60 cups/month	1.02 (0.64-1.63)	0.923	0.92 (0.56-1.52)	0.745
Male				
No drink	1.00		1.00	
1-30 cups/month	0.82 (0.44-1.53)	0.536	1.02 (0.54-1.91)	0.953
30-60 cups/month	0.66 (0.37-1.16)	0.149	0.80 (0.44-1.44)	0.453
> 60 cups/month	0.68 (0.38-1.23)	0.204	1.08 (0.58-2.01)	0.814
Female				
No drink	1.00		1.00	
1-30 cups/month	0.47 (0.26-0.85)	0.012	0.56 (0.31-1.03)	0.063
30-60 cups/month	0.43 (0.26-0.73)	0.002*	0.56 (0.32-0.96)	0.034*
> 60 cups/month	0.33 (0.16-0.70)	0.004*	0.55 (0.25-1.21)	0.136

* Logistic regression model, Significance at P < 0.05

† The model was adjusted for age, sex, income group, BMI, smoking, alcohol consumption, hypertension, diabetes mellitus, hyperlipidemia, stoke, ischemic heart disease histories, nutritional intake (total calories, protein, fat, and carbohydrate intake), and physical exercise.

Table S8. Crude and adjusted odd ratios (95% confidence interval) of coffee drinking habit for thyroid cancer histories

Type of cancer	OR (95% CI) for thyroid cancer			
	Crude	P-value	Adjusted†	P-value
Age < 53 years old				
No drink	1.00		1.00	
1-30 cups/month	0.91 (0.73-1.14)	0.419	0.95 (0.76-1.19)	0.667
30-60 cups/month	0.72 (0.58-0.89)	0.002*	0.75 (0.60-0.93)	0.009*
> 60 cups/month	0.51 (0.40-0.64)	<0.001*	0.68 (0.53-0.87)	0.002*
Age ≥ 53 years old				
No drink	1.00		1.00	
1-30 cups/month	0.85 (0.69-1.05)	0.185	0.87 (0.70-1.08)	0.201
30-60 cups/month	0.76 (0.62-0.92)	0.005*	0.80 (0.65-0.98)	0.029*
> 60 cups/month	0.54 (0.42-0.71)	<0.001*	0.76 (0.57-1.00)	0.054
Male				
No drink	1.00		1.00	
1-30 cups/month	0.73 (0.42-1.28)	0.267	0.78 (0.44-1.38)	0.400
30-60 cups/month	0.64 (0.38-1.06)	0.083	0.70 (0.41-1.18)	0.176
> 60 cups/month	0.55 (0.32-0.95)	0.032*	0.74 (0.42-1.31)	0.302
Female				
No drink	1.00		1.00	
1-30 cups/month	0.96 (0.82-1.13)	0.645	0.93 (0.79-1.10)	0.395
30-60 cups/month	0.81 (0.70-0.94)	0.007*	0.78 (0.67-0.91)	0.001*
> 60 cups/month	0.72 (0.60-0.87)	0.001*	0.70 (0.57-0.85)	<0.001*

* Logistic regression model, Significance at P < 0.05

† The model was adjusted for age, sex, income group, BMI, smoking, alcohol consumption, hypertension, diabetes mellitus, hyperlipidemia, stoke, ischemic heart disease histories, nutritional intake (total calories, protein, fat, and carbohydrate intake), and physical exercise.

Table S9. Crude and adjusted odd ratios (95% confidence interval) of coffee drinking habit for prostate cancer histories

Type of cancer	OR (95% CI) for prostate cancer			
	Crude	P-value	Adjusted†	P-value
Age < 53 years old				
No drink	1.00		1.00	
1-30 cups/month	N/A		N/A	
30-60 cups/month	N/A		N/A	
> 60 cups/month	N/A		N/A	
Age ≥ 53 years old				
No drink	1.00		1.00	
1-30 cups/month	0.88 (0.55-1.42)	0.604	0.96 (0.59-1.56)	0.870
30-60 cups/month	0.79 (0.51-1.22)	0.278	0.86 (0.55-1.35)	0.519
> 60 cups/month	0.62 (0.38-1.02)	0.058	0.83 (0.49-1.39)	0.477
Male				
No drink	1.00		1.00	
1-30 cups/month	0.84 (0.52-1.35)	0.464	0.98 (0.61-1.60)	0.946
30-60 cups/month	0.74 (0.48-1.14)	0.173	0.87 (0.55-1.36)	0.528
> 60 cups/month	0.49 (0.30-0.80)	0.004*	0.86 (0.52-1.44)	0.578
Female				
No drink	1.00		1.00	
1-30 cups/month	N/A		N/A	
30-60 cups/month	N/A		N/A	
> 60 cups/month	N/A		N/A	

* Logistic regression model, Significance at P < 0.05

† The model was adjusted for age, sex, income group, BMI, smoking, alcohol consumption, hypertension, diabetes mellitus, hyperlipidemia, stoke, ischemic heart disease histories, nutritional intake (total calories, protein, fat, and carbohydrate intake), and physical exercise.

Table S10. Crude and adjusted odd ratios (95% confidence interval) of coffee drinking habit for bladder cancer histories

Type of cancer	OR (95% CI) for bladder cancer			
	Crude	P-value	Adjusted†	P-value
Age < 53 years old				
No drink	1.00		1.00	
1-30 cups/month	N/A		N/A	
30-60 cups/month	N/A		N/A	
> 60 cups/month	N/A		N/A	
Age ≥ 53 years old				
No drink	1.00		1.00	
1-30 cups/month	0.52 (0.25-1.09)	0.082	0.51 (0.24-1.09)	0.082
30-60 cups/month	0.91 (0.52-1.61)	0.750	0.86 (0.48-1.55)	0.617
> 60 cups/month	1.34 (0.72-2.49)	0.360	0.98 (0.50-1.92)	0.952
Male				
No drink	1.00		1.00	
1-30 cups/month	0.76 (0.34-1.70)	0.505	0.89 (0.39-1.99)	0.770
30-60 cups/month	0.91 (0.45-1.83)	0.791	1.03 (0.51-2.10)	0.934
> 60 cups/month	0.86 (0.42-1.76)	0.679	1.15 (0.54-2.45)	0.726
Female				
No drink	1.00		1.00	
1-30 cups/month	0.60 (0.21-1.72)	0.338	0.84 (0.29-2.45)	0.752
30-60 cups/month	0.64 (0.26-1.60)	0.340	1.09 (0.42-2.81)	0.859
> 60 cups/month	0.40 (0.11-1.51)	0.178	0.84 (0.21-3.41)	0.809

* Logistic regression model, Significance at P < 0.05

† The model was adjusted for age, sex, income group, BMI, smoking, alcohol consumption, hypertension, diabetes mellitus, hyperlipidemia, stoke, ischemic heart disease histories, nutritional intake (total calories, protein, fat, and carbohydrate intake), and physical exercise.

Table S11. Crude and adjusted odd ratios (95% confidence interval) of physical exercise for gastric cancer histories

Type of cancer	OR (95% CI) for gastric cancer			
	Crude	P-value	Adjusted†	P-value
Age < 53 years old				
No regular exercise	1.00		1.00	
< 150 min/week	0.99 (0.69-1.41)	0.945	0.94 (0.65-1.35)	0.741
≥ 150 min/week	1.32 (1.02-1.72)	0.036*	1.23 (0.94-1.61)	0.125
Age ≥ 53 years old				
No regular exercise	1.00		1.00	
< 150 min/week	1.06 (0.84-1.34)	0.624	1.06 (0.84-1.35)	0.611
≥ 150 min/week	1.24 (1.06-1.45)	0.008*	1.15 (0.98-1.35)	0.092
Male				
No drink	1.00		1.00	
No regular exercise	0.96 (0.74-1.25)	0.762	1.13 (0.87-1.47)	0.376
< 150 min/week	1.31 (1.10-1.58)	0.003*	1.19 (0.99-1.44)	0.066
Female				
No regular exercise	1.00		1.00	
< 150 min/week	0.91 (0.67-1.24)	0.559	0.92 (0.67-1.25)	0.578
≥ 150 min/week	1.21 (0.99-1.48)	0.067	1.18 (0.96-1.45)	0.115

* Logistic regression model, Significance at P < 0.05

† The model was adjusted for age, sex, income group, BMI, smoking, alcohol consumption, hypertension, diabetes mellitus, hyperlipidemia, stoke, ischemic heart disease histories, nutritional intake (total calories, protein, fat, and carbohydrate intake), and coffee consumption.

Table S12. Crude and adjusted odd ratios (95% confidence interval) of physical exercise for hepatic cancer histories

Type of cancer	OR (95% CI) for hepatic cancer			
	Crude	P-value	Adjusted†	P-value
Age < 53 years old				
No regular exercise	1.00		1.00	
< 150 min/week	0.46 (0.10-2.02)	0.304	0.42 (0.10-1.88)	0.260
≥ 150 min/week	1.76 (0.85-3.65)	0.128	1.67 (0.79-3.51)	0.178
Age ≥ 53 years old				
No regular exercise	1.00		1.00	
< 150 min/week	1.16 (0.66-2.04)	0.599	1.16 (0.66-2.05)	0.607
≥ 150 min/week	1.17 (0.79-1.74)	0.426	1.06 (0.71-1.59)	0.777
Male				
No regular exercise	1.00		1.00	
< 150 min/week	0.54 (0.26-1.16)	0.115	0.63 (0.29-1.35)	0.231
≥ 150 min/week	1.35 (0.89-2.06)	0.160	1.22 (0.79-1.89)	0.359
Female				
No regular exercise	1.00		1.00	
< 150 min/week	1.60 (0.77-3.36)	0.209	1.70 (0.81-3.58)	0.162
≥ 150 min/week	1.11 (0.60-2.06)	0.746	1.09 (0.58-2.05)	0.782

* Logistic regression model, Significance at P < 0.05

† The model was adjusted for age, sex, income group, BMI, smoking, alcohol consumption, hypertension, diabetes mellitus, hyperlipidemia, stoke, ischemic heart disease histories, nutritional intake (total calories, protein, fat, and carbohydrate intake), and coffee consumption.

Table S13. Crude and adjusted odd ratios (95% confidence interval) of physical exercise for colon cancer histories

Type of cancer	OR (95% CI) for colon cancer			
	Crude	P-value	Adjusted†	P-value
Age < 53 years old				
No regular exercise	1.00		1.00	
< 150 min/week	0.70 (0.38-1.31)	0.266	0.66 (0.35-1.24)	0.197
≥ 150 min/week	1.50 (1.02-2.19)	0.040*	1.42 (0.96-2.10)	0.080
Age ≥ 53 years old				
No regular exercise	1.00		1.00	
< 150 min/week	1.04 (0.74-1.46)	0.815	1.03 (0.73-1.44)	0.885
≥ 150 min/week	1.71 (1.39-2.11)	<0.001*	1.55 (1.25-1.92)	<0.001*
Male				
No regular exercise	1.00		1.00	
< 150 min/week	0.75 (0.50-1.14)	0.185	0.84 (0.55-1.28)	0.411
≥ 150 min/week	1.66 (1.29-2.14)	<0.001*	1.39 (1.07-1.81)	0.013*
Female				
No regular exercise	1.00		1.00	
< 150 min/week	0.99 (0.65-1.51)	0.962	1.01 (0.66-1.54)	0.966
≥ 150 min/week	1.73 (1.33-2.25)	<0.001*	1.65 (1.26-2.15)	<0.001*

* Logistic regression model, Significance at P < 0.05

† The model was adjusted for age, sex, income group, BMI, smoking, alcohol consumption, hypertension, diabetes mellitus, hyperlipidemia, stoke, ischemic heart disease histories, nutritional intake (total calories, protein, fat, and carbohydrate intake), and coffee consumption.

Table S14. Crude and adjusted odd ratios (95% confidence interval) of physical exercise for breast cancer histories

Type of cancer	OR (95% CI) for breast cancer			
	Crude	P-value	Adjusted†	P-value
Age < 53 years old				
No regular exercise	1.00		1.00	
< 150 min/week	1.06 (0.82-1.37)	0.637	1.12 (0.87-1.45)	0.377
≥ 150 min/week	1.48 (1.23-1.78)	<.0001	1.38 (1.15-1.67)	0.001*
Age ≥ 53 years old				
No regular exercise	1.00		1.00	
< 150 min/week	1.21 (0.94-1.57)	0.146	1.26 (0.97-1.64)	0.078
≥ 150 min/week	1.51 (1.26-1.79)	<0.001*	1.63 (1.36-1.94)	<0.001*
Male				
No regular exercise	1.00		1.00	
< 150 min/week	N/A		N/A	
≥ 150 min/week	N/A		N/A	
Female				
No regular exercise	1.00		1.00	
< 150 min/week	1.26 (1.05-1.51)	0.013*	1.21 (1.01-1.45)	0.043*
≥ 150 min/week	1.61 (1.42-1.83)	<0.001*	1.53 (1.35-1.74)	<0.001*

* Logistic regression model, Significance at P < 0.05

† The model was adjusted for age, sex, income group, BMI, smoking, alcohol consumption, hypertension, diabetes mellitus, hyperlipidemia, stoke, ischemic heart disease histories, nutritional intake (total calories, protein, fat, and carbohydrate intake), and coffee consumption.

Table S15. Crude and adjusted odd ratios (95% confidence interval) of physical exercise for uterine cervix cancer histories

Type of cancer	OR (95% CI) for uterine cervix cancer			
	Crude	P-value	Adjusted†	P-value
Age < 53 years old				
No regular exercise	1.00		1.00	
< 150 min/week	0.95 (0.67-1.34)	0.775	1.07 (0.76-1.51)	0.706
≥ 150 min/week	1.08 (0.83-1.40)	0.579	1.07 (0.82-1.39)	0.624
Age ≥ 53 years old				
No regular exercise	1.00		1.00	
< 150 min/week	0.92 (0.68-1.25)	0.598	1.05 (0.77-1.42)	0.772
≥ 150 min/week	0.90 (0.73-1.11)	0.306	1.05 (0.85-1.30)	0.659
Male				
No regular exercise	1.00		1.00	
< 150 min/week	N/A		N/A	
≥ 150 min/week	N/A		N/A	
Female				
No regular exercise	1.00		1.00	
< 150 min/week	1.02 (0.81-1.28)	0.870	1.06 (0.84-1.33)	0.637
≥ 150 min/week	1.06 (0.90-1.25)	0.500	1.06 (0.90-1.25)	0.497

* Logistic regression model, Significance at $P < 0.05$

† The model was adjusted for age, sex, income group, BMI, smoking, alcohol consumption, hypertension, diabetes mellitus, hyperlipidemia, stoke, ischemic heart disease histories, nutritional intake (total calories, protein, fat, and carbohydrate intake), and coffee consumption.

Table S16. Crude and adjusted odd ratios (95% confidence interval) of physical exercise for lung cancer histories

Type of cancer	OR (95% CI) for lung cancer			
	Crude	P-value	Adjusted†	P-value
Age < 53 years old				
No regular exercise	1.00		1.00	
< 150 min/week	2.25 (0.86-5.92)	0.100	2.23 (0.84-5.93)	0.108
≥ 150 min/week	1.15 (0.44-3.02)	0.776	0.99 (0.37-2.64)	0.986
Age ≥ 53 years old				
No regular exercise	1.00		1.00	
< 150 min/week	0.88 (0.52-1.49)	0.634	0.89 (0.52-1.52)	0.679
≥ 150 min/week	1.28 (0.93-1.78)	0.136	1.14 (0.82-1.60)	0.430
Male				
No regular exercise	1.00		1.00	
< 150 min/week	0.65 (0.34-1.26)	0.205	0.81 (0.42-1.58)	0.541
≥ 150 min/week	1.18 (0.79-1.78)	0.419	0.97 (0.63-1.47)	0.873
Female				
No regular exercise	1.00		1.00	
< 150 min/week	1.43 (0.76-2.70)	0.271	1.47 (0.77-2.79)	0.240
≥ 150 min/week	1.52 (0.95-2.44)	0.082	1.41 (0.87-2.26)	0.163

* Logistic regression model, Significance at P < 0.05

† The model was adjusted for age, sex, income group, BMI, smoking, alcohol consumption, hypertension, diabetes mellitus, hyperlipidemia, stoke, ischemic heart disease histories, nutritional intake (total calories, protein, fat, and carbohydrate intake), and coffee consumption.

Table S17. Crude and adjusted odd ratios (95% confidence interval) of physical exercise for thyroid cancer histories

Type of cancer	OR (95% CI) for thyroid cancer			
	Crude	P-value	Adjusted†	P-value
Age < 53 years old				
No regular exercise	1.00		1.00	
< 150 min/week	1.06 (0.85-1.31)	0.617	1.11 (0.90-1.38)	0.334
≥ 150 min/week	1.44 (1.23-1.69)	<0.001*	1.36 (1.16-1.60)	<0.001*
Age ≥ 53 years old				
No regular exercise	1.00		1.00	
< 150 min/week	1.09 (0.85-1.40)	0.511	1.05 (0.82-1.35)	0.693
≥ 150 min/week	1.44 (1.23-1.70)	<0.001*	1.43 (1.21-1.69)	<0.001*
Male				
No regular exercise	1.00		1.00	
< 150 min/week	1.05 (0.61-1.80)	0.860	0.91 (0.53-1.58)	0.746
≥ 150 min/week	1.39 (0.94-2.05)	0.095	1.10 (0.74-1.64)	0.625
Female				
No regular exercise	1.00		1.00	
< 150 min/week	1.18 (0.99-1.40)	0.063	1.12 (0.94-1.33)	0.197
≥ 150 min/week	1.52 (1.35-1.71)	<0.001*	1.45 (1.29-1.64)	<0.001*

* Logistic regression model, Significance at P < 0.05

† The model was adjusted for age, sex, income group, BMI, smoking, alcohol consumption, hypertension, diabetes mellitus, hyperlipidemia, stoke, ischemic heart disease histories, nutritional intake (total calories, protein, fat, and carbohydrate intake), and coffee consumption.

Table S18. Crude and adjusted odd ratios (95% confidence interval) of physical exercise for prostate cancer histories

Type of cancer	OR (95% CI) for prostate cancer			
	Crude	P-value	Adjusted†	P-value
Age < 53 years old				
No regular exercise	1.00		1.00	
< 150 min/week	N/A		N/A	
≥ 150 min/week	N/A		N/A	
Age ≥ 53 years old				
No regular exercise	1.00		1.00	
< 150 min/week	1.70 (1.04-2.79)	0.034*	1.57 (0.95-2.58)	0.077
≥ 150 min/week	2.30 (1.63-3.24)	<0.001*	1.64 (1.16-2.34)	0.006*
Male				
No regular exercise	1.00		1.00	
< 150 min/week	1.29 (0.80-2.10)	0.294	1.56 (0.96-2.55)	0.075
≥ 150 min/week	2.15 (1.53-3.02)	<0.001*	1.61 (1.13-2.28)	0.008*
Female				
No regular exercise	1.00		1.00	
< 150 min/week	N/A		N/A	
≥ 150 min/week	N/A		N/A	

* Logistic regression model, Significance at P < 0.05

† The model was adjusted for age, sex, income group, BMI, smoking, alcohol consumption, hypertension, diabetes mellitus, hyperlipidemia, stoke, ischemic heart disease histories, nutritional intake (total calories, protein, fat, and carbohydrate intake), and coffee consumption.

Table S19. Crude and adjusted odd ratios (95% confidence interval) of physical exercise for bladder cancer histories

Type of cancer	OR (95% CI) for bladder cancer			
	Crude	P-value	Adjusted†	P-value
Age < 53 years old				
No regular exercise	1.00		1.00	
< 150 min/week	2.41 (0.84-6.96)	0.103	2.06 (0.70-6.04)	0.189
≥ 150 min/week	1.23 (0.43-3.55)	0.698	1.10 (0.37-3.22)	0.868
Age ≥ 53 years old				
No regular exercise	1.00		1.00	
< 150 min/week	0.89 (0.43-1.85)	0.764	0.92 (0.44-1.91)	0.818
≥ 150 min/week	1.23 (0.78-1.94)	0.373	1.10 (0.69-1.75)	0.698
Male				
No regular exercise	1.00		1.00	
< 150 min/week	1.12 (0.56-2.26)	0.745	1.33 (0.66-2.69)	0.432
≥ 150 min/week	1.62 (0.99-2.66)	0.055	1.40 (0.84-2.33)	0.193
Female				
No regular exercise	1.00		1.00	
< 150 min/week	0.86 (0.29-2.53)	0.777	0.95 (0.32-2.84)	0.932
≥ 150 min/week	0.52 (0.21-1.31)	0.167	0.54 (0.21-1.37)	0.193

* Logistic regression model, Significance at P < 0.05

† The model was adjusted for age, sex, income group, BMI, smoking, alcohol consumption, hypertension, diabetes mellitus, hyperlipidemia, stoke, ischemic heart disease histories, nutritional intake (total calories, protein, fat, and carbohydrate intake), and coffee consumption.

Table S20. Crude and adjusted odd ratios (95% confidence interval) of physical exercise for thyroid cancer histories

Type of cancer	OR (95% CI) for thyroid cancer			
	Crude	P-value	Adjusted†	P-value
Age < 53 years old				
No regular exercise	1.00		1.00	
< 150 min/week	1.06 (0.85-1.31)	0.617	1.11 (0.90-1.38)	0.334
≥ 150 min/week	1.44 (1.23-1.69)	<0.001*	1.36 (1.16-1.60)	<0.001*
Age ≥ 53 years old				
No regular exercise	1.00		1.00	
< 150 min/week	1.09 (0.85-1.40)	0.511	1.05 (0.82-1.35)	0.693
≥ 150 min/week	1.44 (1.23-1.70)	<0.001*	1.43 (1.21-1.69)	<0.001*
Male				
No regular exercise	1.00		1.00	
< 150 min/week	1.05 (0.61-1.80)	0.860	0.91 (0.53-1.58)	0.746
≥ 150 min/week	1.39 (0.94-2.05)	0.095	1.10 (0.74-1.64)	0.625
Female				
No regular exercise	1.00		1.00	
< 150 min/week	1.18 (0.99-1.40)	0.063	1.12 (0.94-1.33)	0.197
≥ 150 min/week	1.52 (1.35-1.71)	<0.001*	1.45 (1.29-1.64)	<0.001*

* Logistic regression model, Significance at P < 0.05

† The model was adjusted for age, sex, income group, BMI, smoking, alcohol consumption, hypertension, diabetes mellitus, hyperlipidemia, stoke, ischemic heart disease histories, nutritional intake (total calories, protein, fat, and carbohydrate intake), and coffee consumption.

Table S21. The interaction between coffee consumption, and physical exercise for various cancer histories.

Type of cancer	OR (95% CI) of Interaction	
	Crude	Adjusted†
Gastric cancer	1.02 (0.99-1.05)	1.02 (0.99-1.06)
Hepatic cancer	0.94 (0.86-1.03)	0.93 (0.85-1.02)
Colon cancer	1.03 (0.99-1.08)	1.02 (0.98-1.07)
Breast cancer ‡	1.00 (0.97-1.03)	1.02 (0.98-1.05)
Uterine cervix cancer ‡	1.00 (0.96-1.04)	1.02 (0.98-1.06)
Lung cancer	1.01 (0.94-1.09)	0.99 (0.92-1.07)
Thyroid cancer	1.02 (1.00-1.05)	1.04 (1.02-1.07)
Prostate cancer ‡	1.08 (1.01-1.16)	1.06 (0.99-1.14)
Bladder cancer	1.10 (1.00-1.21)	1.07 (0.97-1.17)

* Logistic regression model, Significance at $P < 0.05$

† The model was adjusted for age, sex, income group, BMI, smoking, alcohol consumption, hypertension, diabetes mellitus, hyperlipidemia, stoke, ischemic heart disease histories, and nutritional intake (total calories, protein, fat, and carbohydrate intake)

‡ Breast and uterine cervix cancer was calculated in Female, and prostate cancer was in Male.

Table S22. The association between physical exercise and thyroid cancer in the subgroup analyses by coffee consumption

Subgroup by coffee consumption	OR (95% CI) of physical exercise for thyroid cancer			
	Crude	P-value	Adjusted†	P-value
No drink (n = 27,131)				
No regular exercise	1.00		1.00	
< 150 min/week	1.10 (0.77-1.57)	0.591	1.09 (0.76-1.56)	0.641
≥ 150 min/week	1.32 (1.04-1.69)	0.025*	1.31 (1.03-1.68)	0.031*
1-30 cups/month (n = 36,235)				
No regular exercise	1.00		1.00	
< 150 min/week	1.10 (0.82-1.49)	0.518	1.15 (0.85-1.56)	0.361
≥ 150 min/week	1.29 (1.03-1.61)	0.029*	1.31 (1.04-1.65)	0.021*
30-60 cups/month (n = 62,806)				
No regular exercise	1.00		1.00	
< 150 min/week	0.89 (0.67-1.19)	0.442	0.93 (0.69-1.24)	0.616
≥ 150 min/week	1.54 (1.28-1.84)	<0.001*	1.58 (1.32-1.90)	<0.001*
> 60 cups/month (n = 36,048)				
No regular exercise	1.00		1.00	
< 150 min/week	1.30 (0.88-1.92)	0.182	1.40 (0.95-2.07)	0.090
≥ 150 min/week	1.36 (1.01-1.82)	0.042*	1.35 (1.01-1.82)	0.040*

* Logistic regression model, Significance at $P < 0.05$

† The model was adjusted for age, sex, income group, BMI, smoking, alcohol consumption, hypertension, diabetes mellitus, hyperlipidemia, stoke, ischemic heart disease histories, and nutritional intake (total calories, protein, fat, and carbohydrate intake)

Table S23. The association between coffee consumption and thyroid cancer in the subgroup analyses by physical exercise

Subgroup by physical exercise	OR (95% CI) of coffee consumption for thyroid cancer			
	Crude	P-value	Adjusted†	P-value
No regular exercise (n = 82,095)				
No drink	1.00		1.00	
1-30 cups/month	0.91 (0.72-1.15)	0.043	0.93 (0.73-1.17)	0.542
30-60 cups/month	0.73 (0.58-0.90)	0.003*	0.72 (0.58-0.91)	0.005*
> 60 cups/month	0.54 (0.42-0.69)	<0.001*	0.66 (0.50-0.87)	0.003*
< 150 min/week (n = 23,667)				
No drink	1.00		1.00	
1-30 cups/month	0.91 (0.61-1.36)	0.652	0.94 (0.63-1.42)	0.773
30-60 cups/month	0.59 (0.39-0.88)	0.010*	0.62 (0.41-0.94)	0.025*
> 60 cups/month	0.63 (0.40-1.00)	0.051	0.87 (0.54-1.42)	0.576
≥ 150 min/week (n = 56,458)				
No drink	1.00		1.00	
1-30 cups/month	0.89 (0.70-1.12)	0.320	0.90 (0.71-1.15)	0.410
30-60 cups/month	0.84 (0.68-1.04)	0.117	0.87 (0.70-1.09)	0.233
> 60 cups/month	0.55 (0.42-0.73)	<0.001*	0.71 (0.53-0.95)	0.023*

* Logistic regression model, Significance at $P < 0.05$

† The model was adjusted for age, sex, income group, BMI, smoking, alcohol consumption, hypertension, diabetes mellitus, hyperlipidemia, stoke, ischemic heart disease histories, and nutritional intake (total calories, protein, fat, and carbohydrate intake)
